# Supplementary material for: Molecular shifts in limb identity underlie development of feathered feet in two domestic avian species
Source: eLife. 2016 Mar 15;5:e12115. doi: 10.7554/eLife.12115 (PMC4805547; doi:10.7554/eLife.12115)
Supplement: Supplementary file 1. — List of oligonucleotide names and targets for genotyping of indel on scaffold 79, qRT-PCR for gene expression assays, in situ hybridization probes, and pyrosequencing for allele specific expression assays. DOI: http://dx.doi.org/10.7554/eLife.12115.020 [file elife-12115-supp1.docx]

**Supplementary File 1. Primers used in this study**

| **Target** | **Primer sequence (5’ to 3’)** |
| --- | --- |
| **Genotyping indel on scaffold 79** |  |
| F primer located 5’ of deletion | ACAGAAAACAAAACCGGCA |
| R primer located within unique indel sequence of feathered allele | TATAAATGCACGCCATCTCG |
| R primer located within deletion | CTGACAGGCACCATGACAAT |
| **qRT-PCR** |  |
| *Actb (C. livia* and *G. gallus)* | CCCAAAGCCAACAGAGAGAA |
|  | ACCAGAGGCATACAGGGACA |
| *H2afy* | ccttcaattggtagtggcaga |
|  | gaagaggacattgttgacacaaa |
| *Pitx1 (C. livia)* | CGTTTGGACCAACCTCACC |
|  | ACAGGTCCATCTGCTGGTTC |
| *Pitx1 (G. gallus)* | accgctaccccgatatgag |
|  | tcccgctttctccacttg |
| *Smad5* | cgagaacactagacgacatattgg |
|  | gctgctgtcacttaaacactcag |
| *Tbx3* (*C. livia*) | gcacctccagcctcaaag |
|  | tcgtctttgctgtctgcatc |
| *Tbx3* (*G. gallus*) | gcacctccagcctcaaag |
|  | gggtcgtctttgctgtcg |
| *Tbx5* (*C. livia*) | ccaacccctaccccatct |
|  | accactgcaccaagagaaaag |
| *Tbx5* (*G. gallus*) | ccaacccctacccgatct |
|  | ggtggtggaacattcctcat |
| *Tgfbi* | ccaaggggaactgaacaaac |
|  | ctccctgcatggacttcag |
| ***in situ* probe template** |  |
| F primer for *Pitx1*, T3 sequence underlined | CGATGATTAACCCTCACTAAAGGGAGAACCAGCAGATGGACCTGT |
| R primer for *Pitx1,* T7 sequence underlined | CGATGTTAATACGACTCACTAGAGGGGGGCTTTGCAAACTGCTGTA |
| F primer for *Tbx5,* T3 sequence underlined | CGATGATTAACCCTCACTAAAGGGAGATGTCCCGGATGCAGAGTA |
| R primer for *Tbx5,* T7 sequence underlined | CGATGTTAATACGACTCACTATAGGGCTGTTTGAGGTCCACACTGC |
| ***Pitx1* SNP pyrosequencing assay** |  |
| F1 | AAATAAAAGCAAACCAACGACG |
| R1 (biotinylated) | ATTGCAACTCATCCATGTACCA |
| Sequencing primer | CAAACCAACGACGCA |
| Sequence to analyze | AC/ACAACAAAACAGCGCTTAAAAATATT |
| ***Tbx3* SNP pyrosequencing assay** |  |
| F1 | GATCAGTTAGTGGGGGCTGCA |
| R1 (biotinylated) | GATGGGCTGCTGCCTGGT |
| Sequencing primer | TGCAGAGACTGGTATCC |
| Sequence to analyze | CG/CTTTTCTTCCCTGGGTCACCAGGCAG |
| ***Tbx5 (C. livia)* SNP pyrosequencing assay** |  |
| F1 (biotinylated) | TTGCAGCTGATGTCTTCTAGGC |
| R1 | CACCGAGCATCCCTACAAGAA |
| Sequencing primer | ACTTCATACAGGACTGAATC |
| Sequence to analyze | C/TGCCCAGCGCCAAGCATGTATGTATG |
| ***Tbx5 (G. gallus)* SNP pyrosequencing assay** |  |
| F1 (biotinylated) | CTTCTGCACCCATGTCTTCC |
| R1 | GTGGTTTTGGTAGGAGGTAACAGC |
| Sequencing primer | GGTAACAGCGATGAAGG |
| Sequence to analyze | CA/GGTCTCG/TGGGAAGACATGGGTGCAGA |
